# Randomized controlled pilot study of a SystemCHANGE™ weight management intervention in stroke survivors: rationale and protocol

**DOI:** 10.1186/1745-6215-14-130

**Published:** 2013-05-07

**Authors:** Matthew Plow, Shirley M Moore, John P Kirwan, Fredrick Frost, Irene Katzan, Sue Jaeger, Jay Alberts

**Affiliations:** 1 Department of Biomedical Engineering, Physical Medicine and Rehabilitation; Cleveland Clinic Lerner Research Institute, 9500 Euclid Ave, ND-20, Cleveland, OH, 44195, USA; 2 The Edward and Louise Mellen Professor of Nursing and Associate Dean for Research; Frances Payne Bolton School of Nursing, Case Western Reserve University, 10900 Euclid Ave, Cleveland, OH, 44106, USA; 3 Department of Pathobiology, Cleveland Clinic Lerner Research Institute, Cleveland, OH, 44195, USA; 4 Department Chair, Physical Medicine and Rehabilitation, Cleveland Clinic Neurological Institute, Cleveland, OH, 44195, USA; 5 Neurological Institute Center for Outcomes Research and Evaluation, Cleveland Clinic, Cleveland, OH, 44195, USA; 6 Cerebrovascular Center, Cleveland Clinic Neurological Institute, Cleveland, OH, 44195, USA; 7 Department of Biomedical Engineering, Center for Neurological Restoration; Cleveland Clinic Lerner Research Institute, 9500 Euclid Ave, ND-20, Cleveland, OH, 44195, USA

**Keywords:** Stroke, Weight management, Obesity, Self-management, Exercise, Physical activity, Nutrition, Diet, Sleep hygiene

## Abstract

**Background:**

Over 65% of stroke survivors are either overweight or obese and have multiple cardiovascular risk factors. However, few studies have examined the effects of comprehensive lifestyle behavior interventions to promote weight loss and control cardiovascular risk factors in stroke survivors. Thus, the purpose of this study is to examine a novel behavior change approach - SystemCHANGE™ - to promote weight loss and improve health and function in stroke survivors. SystemCHANGE™ focuses on redesigning the social environment to achieve a specific goal.

**Methods:**

We will conduct a randomized controlled pilot study to examine the efficacy, feasibility, and safety of the SystemCHANGE™ weight management program in overweight and obese stroke survivors. The central hypothesis of the study is that the SystemCHANGE™ intervention will help overweight and obese stroke survivors lose 5% of their body weight, thereby improving health and function. Thirty-five stroke survivors will be randomized into either the 6-month SystemCHANGE™ intervention or a contact-control intervention. Outcome measures will be assessed at baseline and again at 3 and 6 months after the interventions. Body composition will be assessed using a Bod Pod. Patient-reported outcomes will be the Stroke Impact Scale and Reintegration to Normal Living Index. Objective outcomes will include the 6-Minute Walking Test and Rivermead Motor Assessment.

**Discussion:**

This study will be the first randomized controlled trial to evaluate the efficacy and safety of a weight management intervention in stroke survivors using the SystemCHANGE™ approach. Furthermore, it will be the first empirically-examined comprehensive lifestyle intervention designed to target physical activity, nutrition, and sleep to promote weight loss in stroke survivors.

**Trial registration:**

ClinicalTrials.gov Identifier: NCT01776034

## Background

Over 65% of stroke survivors are either overweight or obese, and a projected 4.98 million stroke survivors in the USA have multiple cardiovascular risk factors [1]. Stroke survivors are at significant risk for de-conditioning [2], elevated inflammatory markers [3], and insulin resistance [4], which subsequently increases risk for secondary stroke and cardiovascular disease [5,6]. Engaging in healthy behaviors, such as physical activity, good sleep hygiene, and nutrition, to facilitate energy balance may help reduce the risk of secondary conditions and ultimately improve physical function and quality of life in stroke survivors [5,7].

However, stroke survivors can experience many barriers to engaging in a healthy lifestyle to achieve energy balance [8]. Mental and physical impairments interacting with a non-supportive environment can create multiple challenges to engagement in healthy behaviors, which can perpetuate a disabling cycle [9]. Specifically, inactivity, unhealthy eating, and poor sleeping habits can lead to obesity. Obesity can increase mobility problems, fatigue, depression, and further difficulties engaging in healthy behavior. Supporting stroke survivors to achieve energy balance and a healthy body weight may be a strategy to counteract the disablement process.

Most studies examining lifestyle interventions in stroke survivors have focused on promoting physical activity and/or teaching self-management skills [10], which only partially addresses behaviors associated with achieving energy balance [11]. Thus, effective strategies to promote weight loss in stroke survivors remain elusive. Research in the general population indicates weight management interventions should target both physical activity and nutrition [11], and that promoting good sleep hygiene may be an additional strategy to facilitate weight loss [12]. Furthermore, research in people with disabling conditions indicates that impairments often create barriers to engagement in healthy behaviors, but that utilizing self-management skills and having ample social support can facilitate overcoming such health-related barriers [13]. Thus, based on research in the general population and in people with disabling conditions, we contend that an effective weight management intervention in stroke survivors should target physical activity, nutrition, sleep, and self-management skills, as well as utilize strategies to foster a supportive social environment.

Shirley Moore recently developed such an intervention approach called SystemCHANGE™ [14], a new behavior change intervention drawing from social ecological theories [15-17]. SystemCHANGE™ focuses on redesigning the environment using trial-and-error ‘experiments’ to achieve a specific goal. SystemCHANGE™ is in contrast to cognitive-behavioral interventions that focus on changing a person’s viewpoint of a situation and increasing motivation. In SystemCHANGE™_,_ individuals are taught a set of skills to assist them in incorporating habitual lifestyle behaviors into their daily systems so they succeed despite wavering motivation. Strategies include identifying a chain of steps/events that comprise the system in which the desired change is to occur, keeping track of data about the system process to understand it, implementing short trials of possible improvement solutions, evaluating success by reviewing data, and making provisions for holding the gains. Given the novelty of SystemCHANGE™ to focus on an individual’s surrounding environment rather than an individual’s motivation and willpower to change behavior, this approach is now being tested in various population segments (for example, overweight children and patients with HIV). We propose to adapt the SystemCHANGE™ intervention approach to promote weight loss in overweight and obese stroke survivors. This study will represent the first empirically-examined comprehensive lifestyle intervention designed to target physical activity, nutrition, and sleep to promote weight loss in stroke survivors.

The specific aims are to engage stroke survivors and their families as co-designers to adapt the SystemCHANGE™ intervention, and then conduct a randomized controlled pilot study to provide preliminary estimates of the intervention’s efficacy in 35 stroke survivors. Herein, we will focus on describing the methodology for the pilot randomized controlled trial rather than the qualitative methodology. The pilot study will provide effect size estimates on the following primary outcomes: body weight and patient-reported and objective outcomes of health and function. Secondary outcomes include questionnaires on symptoms, healthy behaviors, psychosocial mediators, and biomarkers of cardiovascular risk. The central hypothesis of the study is that the SystemCHANGE™ intervention will help overweight and obese stroke survivors lose 5% of their weight, thereby improving health and function. Hypothesized mechanisms of action will involve significant improvements in healthy behaviors, symptoms, psychosocial constructs, and cardiovascular risk.

## Methods

### Trial design

We will conduct a single-center, randomized, controlled, parallel-group, rater-blinded pilot study. Thirty-five participants will be recruited and randomized into the SystemCHANGE™ intervention or a contact-control information-only intervention. Both interventions will be delivered over a 6-month period. All outcomes will be administered before the interventions and again at 3 and 6 months. The Cleveland Clinic Institutional Review Board approved this study. We will obtain informed consent from each stroke survivor that participates in the study.

### Participants

The following methods will be used for recruitment: (1) fliers distributed at support groups; (2) referrals from clinicians; and (3) medical chart reviews. Individuals will undergo a three-part screening to confirm eligibility (phone, physician, and in-person). Subjects who meet the eligibility requirements over the phone will be mailed a demographic sheet and a release form to contact their physician. Once we confirm eligibility from their physician, arrangements will be made to re-confirm eligibility in person, obtain informed consent, collect pre-test data, and randomize participants into the SystemCHANGE™ intervention or the control intervention. Main inclusion criteria are: (1) a physician-confirmed unilateral ischemic or hemorrhagic stroke (at least 6 weeks post-stroke); (2) physician consent to participate; (3) age 30 to 75 years; (4) a BMI between 25 to 40 kg/m^2^; and (5) a stable weight for the past 4 weeks. Individuals will be excluded if they have: (1) heart disease (that is, myocardial infarction, congestive heart failure, coronary artery bypass grafting, or valve replacement during the past 3 months, serious cardiac arrhythmias, hypertrophic cardiomyopathy, and severe aortic stenosis); (2) pulmonary embolus; (3) uncontrolled resting blood pressure (that is, 140/90 mmHg) or abnormal blood pressure or heart rate response during the 6-Minute Walking Test [18] (for example, drop in systolic blood pressure of >10 mmHg from baseline), (4) uncontrolled diabetes (hospitalized in the past 6 months); (5) unable to communicate (for example, severe aphasia); (6) severe cognitive deficits (weighted score <12 on the short version of the Blessed Orientation Memory Concentration test [19]); (7) pregnancy; (8) three or more falls in the past month; (9) inability to walk 3 meters with or without a mobility device; (10) weight loss medications or medications that cause excessive weight gain (for example, corticosteroids and antipsychotic); and (11) gastric bypass surgery.

Study criteria were selected to help ensure patient safety, create a homogeneous sample of mild to moderate impairments, minimize confounders, and avoid ceiling effects. We are excluding individuals >75 years of age to minimize risk of weight loss, muscle atrophy, and functional declines. Individuals with serious heart conditions are being excluded because of the prescription of a physical activity program. We are also excluding people with uncontrolled blood pressure or diabetes because these individuals need medical treatment, which will likely result in prescribing or changing medications that can confound study results.

### Interventions

#### SystemCHANGE™

The SystemCHANGE™ intervention will be delivered over a 6-month period involving 12 face-to-face group sessions (1.5 h each) held weekly for 3 months, followed by 3-monthly ‘booster calls’. Intervention groups include up to 10 patients, and family is encouraged to attend. Intervention sessions consist of 30-min of behavior change activities and 60-min focused on healthy behaviors. Sessions will be led by a health education specialist and held at local community centers. Below we outline each topic area of the SystemCHANGE™ intervention.

#### Diet

The recommended diet will be consistent with the *Dietary Guidelines for Americans 2010* document [20]. Emphasis will be placed on restricting fatty foods and consuming more fruits and vegetables. Total recommended daily caloric intake will be consistent with recommended degrees of weight reduction, based on a graded reduction in total daily caloric intake adjusted to meet goals and avoid rapid weight loss. Each participant will receive a personalized diet plan based on their food diary.

#### Physical activity

The goal will be to increase physical activity levels, reduce sedentary behaviors, and encourage participation in a strength training program. The physical activity program will be based on the patients’ preference and goals. In a graded fashion, we will encourage participants to achieve recommended physical activity guidelines, which will include a strength training program of major muscle groups.

#### Sleep

The sleep component will address recommended sleep duration, the process and stages of sleep, benefits of sleep, and the relationship of sleep to hunger, stress, fatigue, and obesity. Good sleep hygiene and barriers to adequate sleep will be discussed. Strategies will include minimizing procrastination, regularizing a sleep schedule, learning the components of stimulus control therapy, and relaxation strategies.

#### Symptom management

Kate Lorig’s self-management framework [21] will be used to guide the symptom management component. The overall goal of this component will be to provide specific strategies on managing symptoms and to teach participants the necessary skill set to incorporate these strategies into their lives. We will focus on teaching skills related to managing emotions, communicating with caregivers, and managing fatigue (for example, taking rest breaks and re-organizing work spaces) and pain (for example, visualization and breathing exercises).

#### Contact-control intervention

In contrast to the SystemCHANGE™ arm, participants assigned to the information-only group will receive pamphlets that contain information on healthy eating, physical activity, sleep, and symptom management, and will be telephoned at the same time points as the SystemCHANGE™ sessions. These 20-min phone calls are intended to teach participants recommended guidelines for engaging in healthy behaviors and reducing cardiovascular risk. However, the skills taught in the SystemCHANGE™ intervention (for example, identifying a chain of steps/events that comprise the system in which the desired change is to occur) will not be taught in the control intervention. Thus, we will be able to determine whether it is the skills taught in the SystemCHANGE™ intervention or simply the learning of guidelines that promotes engagement in healthy behaviors. Furthermore, the phone calls in the contact-control intervention will provide an equivalent number of contacts with the health education specialist as in the SystemCHANGE™ intervention. Thus, we will be able to determine whether it is the skills taught in the SystemCHANGE™ intervention or simply the interactions with the health education specialist and creating accountability that promotes engagement in healthy behaviors.

#### Booster sessions

In the SystemCHANGE™ intervention, the booster sessions will assist participants to use strategies taught in the face-to-face sessions to manage the maintenance phase of behavior change. The health education specialists will talk to patients about their efforts in self-monitoring and assist them to identify situations that are high risk for relapse. In the contact-control intervention, the booster sessions will be generic and answer questions about engaging in healthy behaviors.

#### Treatment fidelity

To promote standard application of the interventions in both the SystemCHANGE™ group and the control group, a manual of operating procedures and lesson plans outlining each session will be provided to the health education specialist and incorporated into their training. We will use the same person to deliver the interventions in both groups to facilitate standardized delivery. The health education specialist will also be given a checklist to review and use in each session and will be monitored in-person at random intervals to determine the extent to which the lesson plans are followed. Intervention fidelity will be further monitored by tracking participant attendance, monitoring completion of homework assignments, and administering quizzes to assess participants’ understanding of the material.

### Outcomes

Body fat and muscle composition will be assessed using a Bod Pod, which uses air displacement plethysmography to determine body composition [22]. Patient-reported outcomes will be the Stroke Impact Scale [23] and Reintegration to Normal Living Index [24]. Objective outcomes of health and function will be the 6-Minute Walking Test [18] and Rivermead Motor Assessment [25]. Assessors will be blinded to treatment groups.

Secondary outcomes to examine health behavior change will include a 3-day food diary, the Physical Activity and Disability Survey [26], and sleep quality measured with Neuro-QOL [27]. Fatigue, pain, anxiety, and depression will also be measured with Neuro-QOL [27]. Potential psychosocial mediators that will be measured include self-efficacy for diet [28], physical activity [29] and self-management [30], and social support for healthy behaviors [31]. Finally, assays will be performed for cholesterol, triglycerides, C-reactive protein, and hemoglobin A_1c_.

### Sample size

Our goal to recruit 35 stroke survivors is consistent with recommendations in the literature for pilot studies. Hertzog [32] has shown that samples of 10 to 20 participants per group are often adequate for evaluating feasibility in a pilot study. Furthermore, Dobkin [33] has suggested that 15 participants in each research arm are sufficient for determining whether a larger multicenter trial should be conducted.

### Randomized allocation procedure

Allocation will be performed using a permuted block randomization procedure to help ensure a 1:1 ratio in each group. The allocation sequence will be determined using a computerized random number generator with block sizes of four participants. The allocation sequence will be concealed from the health education specialist and researchers who are enrolling potential participants with a sequential number of opaque sealed envelopes. Envelopes will be opened only after all pre-test data is collected. A researcher with no involvement in the delivery of the interventions or the screening of participants will prepare the envelopes.

### Blinding

It will not be possible to blind the health education specialist or participants to group assignment. However, steps will be taken to blind the researchers who administer the outcome measures. Researchers who administer the outcome measures will be different from the researchers who conduct the screenings and deliver the interventions. Participants will be instructed not to tell the assessor of their group assignment.

### Statistical methods

Recruitment and data collection efforts will be managed with Research Electronic Data Capture [34]. The first step in the analysis will be to compute summary statistics, calculate outcome scores, and conduct quality control assessments. Outcome measures will be analyzed using multivariate repeated measures or one-way ANOVAs (assuming data are normally distributed). Between-subject effects will be examined by subtracting baseline scores from post-test scores and utilizing one-way MANOVA to determine whether there are significant improvements in change scores in the SystemCHANGE™ group compared to the contract-control group. Within-subject effects will be examine with repeated-measures MANOVA comparing baseline data immediately before the SystemCHANGE™ intervention to data at 3 and 6 months after receiving the SystemCHANGE™ intervention. To help minimize missing data, we will follow recommendations by Little *et al*. [35] (for example, providing monetary incentives). Effect sizes will be calculated as Cohen’s d. Feasibility and safety of the intervention will be assessed with descriptive statistics (frequencies, percentages, means, and standard deviations): number of contacted *versus* recruited subjects, reasons for non-participation, attrition (with timing and reasons), time required to train health education specialist and arrange follow-up phone calls, and adverse event monitoring (control *versus* treatment group).

### Risk and safety monitoring

The risk level of this protocol is low to moderate due to recruiting overweight and obese patients and the unknown risks of encouraging weight loss in stroke survivors. Participants will be asked to exercise and change their dietary habit, which poses some risk; however, inclusion-exclusion criteria will help minimize these risks. Data on physical function, weight loss, muscle loss, and other health status changes (that is, cardiac, musculoskeletal, or digestive) will be monitored closely.

## Discussion

This pilot study will represent the first randomized controlled trial to evaluate the efficacy and safety of a weight management intervention in stroke survivors using a SystemCHANGE™ intervention approach. The trial is designed to obtain effect size calculations and examine the feasibility and safety of conducting a larger clinical trial. While it will not be possible to conduct a double-blinded study, the implementation of a contact control group will help enhance retention and allow us to determine whether the actual strategies used in the SystemCHANGE™ intervention are more effective in promoting behavior change than contacts with a health education specialist. Using both self-report and objective measures of function and obtaining body composition measurements throughout the study will be important components in monitoring for potential adverse events.

### Obesity paradox

The relationship between nutritional status, disabling conditions, and physical function is complex and not fully understood [36,37]. A recent observational study indicated an ‘obesity paradox’ in stroke survivors [38]; that is, being either overweight or obese was associated with significantly better early and long-term survival rates compared to those with normal BMI. In a review article, Lavie *et al*. [39] noted that obesity is associated with a more favorable prognosis in at least 10 different disabling conditions, such as heart failure, advanced cancers, and HIV. Furthermore, research also indicates that weight loss is related to muscle atrophy, which can subsequently result in functional decline [40]. Thus, the existing literature raises an important question: Will weight loss adversely affect physical function or morbidity in stroke survivors?

Preliminary research in older adults and patients with heart disease or cancer suggest that modest weight loss does not adversely affect health outcomes [39]. It has also been argued that findings of the obesity paradox may be confounded by selection bias and that the use of traditional BMI categories may not be appropriate for people with disabling conditions because it does not account for muscle atrophy [36,39]. In the 2011 study showing the obesity paradox in stroke survivors, Vemmos *et al*. [38] concluded that ‘results cannot support recommendations to overweight or obese patients not to lose weight after acute ischemic stroke. On the contrary, taking into account the numerous studies that linked increased BMI with cardiovascular events, prevention of weight gain should be strongly promoted.’ Clearly, there is a need to move beyond observational studies to randomized controlled trials that can truly determine the effects of weight loss and reductions in percent body fat on health and function. We contend that weight management interventions in stroke survivors should emphasize strength training to help prevent muscle loss and use percent body fat and waist circumference as indicators of success and safety monitoring rather than body weight alone.

### Potential limitations and confounders

Potential limitations and confounders to the study are the following: (1) changes in medication or dose; (2) evaluating a comprehensive lifestyle intervention rather than focusing on changing one particular behavior; and (3) a subgroup of participants may be non-responsive to a behavioral intervention.

(1) An important component of the study will be excluding individuals who are taking medications known to cause extensive weight gain or loss. Medication changes will also be monitored at each assessment time-point and their possible confounding effects will be examined in our preliminary review of the data. We will also contact the patients’ physician to request that changes in medication causing weight gain or loss only occur when medically necessary.

(2) Although it is difficult to determine which components of an intervention (that is, active ingredients) are effective when evaluating a comprehensive lifestyle intervention, we feel it is necessary to implement such an intervention because multiple behaviors influence weight loss (that is, physical activity, nutrition, reduced stress, and adequate sleep), and stroke survivors experience health-related barriers that can be overcome through teaching self-management skills.

(3) Behavioral approaches to weight loss may not be effective for all patients. However, we expect that new SystemCHANGE™ approach will be more effective. Furthermore, we will explore patient characteristics that influence the intervention’s effectiveness. In the trend towards personalized medicine, an important goal of future studies will be to identify those patients who are non-responsive to behavioral interventions and to make recommendations for alternative interventions, such as bariatric surgery.

We lastly note that observational studies indicate that people with disabilities are two to four times more likely to either be overweight or obese [37,41,42]. However, a dearth of research has evaluated intervention strategies promoting energy balance among people with disabilities. Thus, developing and evaluating a weight management intervention for stroke survivors could also help to fill research gaps within the broader disability literature. Furthermore, examining the effects of the SystemCHANGE™ intervention is novel in the disability literature. SystemCHANGE™ focuses on changing the surrounding social environment rather than a person’s motivation to change behavior, which may be particularly relevant for people with disabling conditions who often rely on formal and informal caregivers for tangible social support, such as grocery shopping and meal preparation [43].

## Trial status

The status of the trial was ongoing and actively recruiting stroke survivors at the time that the protocol was submitted for publication.

## Competing interests

The authors declare that they have no competing interests with this particular study.

## Authors’ contributions

Each author has made contributions to the conception, design, or implementation of the study. MP, SM, JA, and JK contributed to the research design. SM designed the intervention and MP adapted the intervention for stroke survivors. FF, SJ, and IK contributed to the implementation of the study. All authors have read and approved the manuscript for submission.

## References

[B1] Kesarwani M Perez A Lopez VA Wong ND Franklin SS Cardiovascular comorbidities and blood pressure control in stroke survivors J Hypertens 2009 27 1056 1063 10.1097/HJH.0b013e32832935ce 19405168

[B2] Ivey FM Macko RF Ryan AS Hafer-Macko CE Cardiovascular health and fitness after stroke Top Stroke Rehabil 2005 12 1 16 15735997 10.1310/GEEU-YRUY-VJ72-LEAR

[B3] Beamer NB Coull BM Clark WM Briley DP Wynn M Sexton G Persistent inflammatory response in stroke survivors Neurology 1998 50 1722 1728 10.1212/WNL.50.6.1722 9633717

[B4] Ivey FM Ryan AS Hafer-Macko CE Garrity BM Sorkin JD Goldberg AP Macko RF High prevalence of abnormal glucose metabolism and poor sensitivity of fasting plasma glucose in the chronic phase of stroke Cerebrovasc Dis 2006 22 368 371 10.1159/000094853 16888377

[B5] Furie KL Kasner SE Adams RJ Albers GW Bush RL Fagan SC Halperin JL Johnston SC Katzan I Kernan WN Mitchell PH Ovbiagele B Palesch YY Sacco RL Schwamm LH Wassertheil-Smoller S Turan TN Wentworth D on behalf of the American Heart Association Stroke Council, Council on Cardiovascular Nursing, Council on Clinical Cardiology, and Interdisciplinary Council on Quality of Care and Outcomes Research Guidelines for the prevention of stroke in patients with stroke or transient ischemic attack: a guideline for healthcare professionals from the American heart Association/American stroke association Stroke 2011 42 227 276 10.1161/STR.0b013e3181f7d043 20966421

[B6] Kopunek SP Michael KM Shaughnessy M Resnick B Nahm ES Whitall J Goldberg A Macko RF Cardiovascular risk in survivors of stroke Am J Prev Med 2007 32 408 412 10.1016/j.amepre.2007.01.021 17478267 PMC1963444

[B7] Roger VL Go AS Lloyd-Jones DM Adams RJ Berry JD Brown TM Carnethon MR Dai S de Simone G Ford ES Fox CS Fullerton HJ Gillespie C Greenlund KJ Hailpern SM Heit JA Ho PM Howard VJ Kissela BM Kittner SJ Lackland DT Lichtman JH Lisabeth LD Makuc DM Marcus GM Marellia A Matchar DB McDermott MM Meigs JB Moy CS Heart disease and stroke statistics–2011 update: a report from the American heart association Circulation 2011 123 e18 e209 10.1161/CIR.0b013e3182009701 21160056 PMC4418670

[B8] Rimmer JH Wang E Smith D Barriers associated with exercise and community access for individuals with stroke J Rehabil Res Dev 2008 45 315 322 10.1682/JRRD.2007.02.0042 18566948

[B9] Rimmer JH Exercise and physical activity in persons aging with a physical disability Phys Med Rehabil Clin N Am 2005 16 41 56 10.1016/j.pmr.2004.06.013 15561544

[B10] Redfern J McKevitt C Wolfe CD Development of complex interventions in stroke care: a systematic review Stroke 2006 37 2410 2419 10.1161/01.STR.0000237097.00342.a9 16902171

[B11] Seagle HM Strain GW Makris A Reeves RS Position of the American Dietetic Association: weight management J Am Diet Assoc 2009 109 330 346 19244669 10.1016/j.jada.2008.11.041

[B12] Taheri S The link between short sleep duration and obesity: we should recommend more sleep to prevent obesity Arch Dis Child 2006 91 881 884 10.1136/adc.2005.093013 17056861 PMC2082964

[B13] Stuifbergen AK Building health promotion interventions for persons with chronic disabling conditions Fam Community Health 2006 29 28S 34S 10.1097/00003727-200601001-00006 16344634

[B14] Webel AR Moore SM Hanson JE Patel SR Schmotzer B Salata RA Improving sleep hygiene behavior in adults living with HIV/AIDS: a randomized control pilot study of the SystemCHANGE™–HIV intervention Appl Nurs Res 2012 pii S0897 1897 (12)00074-2 23265919 10.1016/j.apnr.2012.10.002 PMC3631581

[B15] Sallis JF Cervero RB Ascher W Henderson KA Kraft MK Kerr J An ecological approach to creating active living communities Annu Rev Public Health 2006 27 297 322 10.1146/annurev.publhealth.27.021405.102100 16533119

[B16] Gregson J Foerster SB Orr R Jones L Benedict J Clarke B Hersey J Lewis J Zotz AK System, environmental, and policy changes: using the social-ecological model as a framework for evaluating nutrition education and social marketing programs with low-income audiences J Nutr Educ 2001 Suppl 1 S4 S15 12857540 10.1016/s1499-4046(06)60065-1

[B17] Baranowski T Cullen KW Nicklas T Thompson D Baranowski J Are current health behavioral change models helpful in guiding prevention of weight gain efforts? Obes Res 2003 Suppl 1 23S 43S 14569036 10.1038/oby.2003.222

[B18] Butland RJ Pang J Gross ER Woodcock AA Geddes DM Two-, six-, and 12-minute walking tests in respiratory disease Br Med J (Clin Res Ed) 1982 284 1607 1608 10.1136/bmj.284.6329.1607 PMC1498516 6805625

[B19] Katzman R Brown T Fuld P Peck A Schechter R Schimmel H Validation of a short orientation-memory-concentration test of cognitive impairment A J Psychiatry 1983 140 734 739 10.1176/ajp.140.6.734 6846631

[B20] United States. Dept. of Health and Human Services, United States. Dept. of Agriculture, United States. Dietary Guidelines Advisory Committee Dietary guidelines for Americans, 2010 2010 7 Washington, D.C: G.P.O

[B21] Lorig KR Holman H Self-management education: history, definition, outcomes, and mechanisms Ann Behav Med 2003 26 1 7 12867348 10.1207/S15324796ABM2601_01

[B22] Minderico CS Silva AM Teixeira PJ Sardinha LB Hull HR Fields DA Validity of air-displacement plethysmography in the assessment of body composition changes in a 16-month weight loss program Nutr Metab (Lond) 2006 3 32 10.1186/1743-7075-3-32 16925811 PMC1560140

[B23] Duncan PW Bode RK Min Lai S Perera S Rasch analysis of a new stroke-specific outcome scale: the stroke impact scale Arch Phys Med Rehabil 2003 84 950 963 10.1016/S0003-9993(03)00035-2 12881816

[B24] Wood-Dauphinee S Williams JI Reintegration to normal living as a proxy to quality of life J Chronic Dis 1987 40 491 502 10.1016/0021-9681(87)90005-1 3597654

[B25] Lincoln N Leadbitter D Assessment of motor function in stroke patients Physiotherapy 1979 65 48 51 441189

[B26] Rimmer JH Riley BB Rubin SS A new measure for assessing the physical activity behaviors of persons with disabilities and chronic health conditions: the physical activity and disability survey Am J Health Promot 2001 16 34 42 10.4278/0890-1171-16.1.34 11575054

[B27] Gershon RC Lai JS Bode R Choi S Moy C Bleck T Miller D Peterman A Cella D Neuro-QOL: quality of life item banks for adults with neurological disorders: item development and calibrations based upon clinical and general population testing Qual Life Res 2012 21 475 486 10.1007/s11136-011-9958-8 21874314 PMC3889669

[B28] Sallis J Pinski R Grossman T Nader PR The development of self-efficacy scales for health related diet and exercise behaviors Health Educ Res 1988 3 283 292 10.1093/her/3.3.283

[B29] Marcus BH Selby VC Niaura RS Rossi JS Self-efficacy and the stages of exercise behavior change Res Q Exerc Sport 1992 63 60 66 10.1080/02701367.1992.10607557 1574662

[B30] Lorig KR Sobel DS Ritter PL Laurent D Hobbs M Effect of a self-management program on patients with chronic disease Eff Clin Pract 2001 4 256 262 11769298

[B31] Sallis JF Grossman RM Pinski RB Patterson TL Nader PR The development of scales to measure social support for diet and exercise behaviors Prev Med 1987 16 825 836 10.1016/0091-7435(87)90022-3 3432232

[B32] Hertzog MA Considerations in determining sample size for pilot studies Res Nurs Health 2008 31 180 191 10.1002/nur.20247 18183564

[B33] Dobkin BH Progressive staging of pilot studies to improve phase III trials for motor interventions Neurorehab Neural Repair 2009 23 197 206 10.1177/1545968309331863 PMC4099048 19240197

[B34] Harris PA Taylor R Thielke R Payne J Gonzalez N Conde JG Research electronic data capture (REDCap)–a metadata-driven methodology and workflow process for providing translational research informatics support J Biomed Inform 2009 42 377 381 10.1016/j.jbi.2008.08.010 18929686 PMC2700030

[B35] Little RJ D’Agostino R Cohen ML Dickersin K Emerson SS Farrar JT Frangakis C Hogan JW Molenberghs G Murphy SA Neaton JD Rotnitzky A Scharfstein D Shih WJ Siegel JP Stern H The prevention and treatment of missing data in clinical trials N Engl J Med 2012 367 1355 1360 10.1056/NEJMsr1203730 23034025 PMC3771340

[B36] Rimmer J Wang E Yamaki K Davis B Documenting Disparities in Obesity and Disability Focus: A Publication of the National Center for the Dissemination of Disability Research (NCDDR) 2010 24 1 16

[B37] Liou TH Pi-Sunyer FX Laferrere B Physical disability and obesity Nutr Rev 2005 63 321 331 10.1111/j.1753-4887.2005.tb00110.x 16295145

[B38] Vemmos K Ntaios G Spengos K Savvari P Vemmou A Pappa T Manios E Georgiopoulos G Alevizaki M Association between obesity and mortality after acute first-ever stroke: the obesity-stroke paradox Stroke 2011 42 30 36 10.1161/STROKEAHA.110.593434 21127299

[B39] Lavie CJ Milani RV Ventura HO Obesity and cardiovascular disease: risk factor, paradox, and impact of weight loss J Am Coll Cardiol 2009 53 1925 1932 10.1016/j.jacc.2008.12.068 19460605

[B40] David RT Loss of skeletal muscle mass in aging: examining the relationship of starvation, sarcopenia and cachexia Clin Nutr 2007 26 389 399 10.1016/j.clnu.2007.03.008 17499396

[B41] Rimmer JH Wang E Obesity prevalence among a group of Chicago residents with disabilities Arch Phys Med Rehabil 2005 86 1461 1464 10.1016/j.apmr.2004.10.038 16003681

[B42] Weil E Wachterman M McCarthy EP Davis RB O’Day B Iezzoni LI Wee CC Obesity among adults with disabling conditions JAMA 2002 288 1265 1268 10.1001/jama.288.10.1265 12215134

[B43] Plow M Finlayson M A qualitative study of nutritional behaviors in adults with multiple sclerosis J Neurosci Nurs 2012 44 337 350 10.1097/JNN.0b013e3182682f9b 23124124

